# Auscultatory Percussion

**Published:** 1891-07-25

**Authors:** 


### THE PRACTITIONER'S MIRROR AND RETROSPECT.

[*The Editor will be glad to receive offers of co-operation and contributions from members of the profession. All letters should be addressed to* THE EDITOR, THE LODGE, PORCHESTER SQUARE, LONDON, W.]

### MEDICINE.

### AUSCULTATORY PERCUSSION.

Auscultatory percussion, though not altogether a new method of physical investigation, has been so little practised, that to the great majority of our readers even the name will be entirely new. There is, however, very little doubt that we have in this method a means of arriving at important information as to the condition of certain viscera, and the investigations of Professor Jackson will prove of great service, for they show how far we may rely on the method, especially in diseases of the heart. In his communication to the New York Academy of Medicine* we are reminded that in 1840 Dr. George Cammann and Dr. Alonzo Clark suggested the employment of auscultatory percussion as a valuable aid to diagnosis, and claimed that by this method the heart could be measured in all but its antero-posterior diameter, under most, perhaps all, circumstances of health and disease, with hardly less exactness than if the organ were exposed before us, and that the outlines of the liver, spleen, and kidney can be traced in the same way. The method of practising auscultatory percussion is very simple; the ordinary Cammann stethoscope is held firmly against the integument over the region of the solid organ or tissue to be investigated, while percussion is made outside the periphery of the organ toward the bell of the stethoscope until a point is reached where there is an immediate and very great increase in and a change in the pitch of the percussion sound. This point is regarded as the edge of the solid organ or new growth. Dr. Jackson has for a long time been in the habit of employing this method, and with considerable satisfaction, although he realized that his results were not always exact. In order to test the value of auscultatory percussion, he instituted two series of experiments—one series upon the dead subject, the other upon the living patient—with a view to the determination of the following points:—(1) Is the method superior to ordinary percussion? (2) Is it an accurate method of estimating the size and outline of the liver, the spleen, the kidney, and the heart? (3) What are the errors of this method? (4) What are the conditions producing these errors? (5) Can enlargement of the heart be recognized by auscultatory percussion alone in any given case? He mapped out the supposed outlines of the liver, spleen, and heart in a number of cases just previous to autopsy, and marked the supposed position of the viscera by means of long pins driven through the abdomen or chest. As regards the liver and spleen the results were eminently unsatisfactory; in the course of a number of experiments only *one liver* and *one spleen* were accurately outlined, and examinations of the kidney were equally misleading.

The examination of the heart was much more satisfactory in its results, and was conducted in the following manner:—The small chest piece of the ordinary Cammann binaural stethoscope was pressed very firmly by an assistant over the area of cardiac dulness, or roughly, midway between the nipple and the left edge of the sternum, while the doctor made forcible percussion over the chest, beginning at the apex of the left lung, and percussing downward till the muffled sound suddenly gave place to high-pitched sound of very considerable intensity and of a peculiarly wooden quality; this point, about an inch from the left of the sternum and usually under the third rib, was marked with a dermographic pencil. In a similar manner was indicated the periphery of the heart, usually ending at the second space in the right side. Pins were then introduced in the intercostal spaces in such a way as to correspond as nearly as possible with the marks upon the skin. An exact copy of the outline of the heart was then taken on tracing linen, the position of each pin was carefully noted, the midsternal line was drawn, and the outline of the nipple traced to obtain anatomical data for subsequent measurements. When the autopsy was made, the position of the pins, whether right or wrong, were marked upon the tracing linen. In a number of cases the ordinary percussion dulness was also noted. This latter was usually much less satisfactory than the area obtained by auscultatory percussion; the edges were much less distinct, and often no idea of the area of the heart could be formed. It must be remembered that these outlines represented the heart muscle itself, and had no reference to the pericardial sac, or what is commonly known as the area of cardiac dulness.

Now, as to the result of these experiments. In nineteen examinations the outline of the heart was determined with sufficient accuracy for all practical purposes in one half the number, and in two-thirds of the cases the outline was determined within half an inch of the heart borders, either within or without. In studying the errors to which we are liable, it was found that these errors might be grouped into two sets: (1) Those in which no definite idea could be formed as to the position of the borders of the heart, by reason on the one hand of the excessive intensity of the percussion sound over a large area, and on the other hand, by reason of the indistinct character of the percussion sound; this latter was the case with the right border only. (2) Those in which the sounds seemed fairly clear, but the area of the heart, as mapped out, was incorrect, being either too large or too small.

In both sets of cases the upper border seemed to be invariably correct.

In cases of pleurisy with moderate effusion; in cases of pus, and in cases of serum in the pericardial sac; in cases of complete consolidation of the lung, and in cases of emphysema of the lungs, the area of the heart was correctly recognised, but was erroneously mapped out in cases of pleurisy with extreme effusion, and in some cases of healthy lung and pleuræ. The proximity of the liver also causes error. The great source of error is traceable, in Dr. Jackson's opinion, to the resonance which is transmitted from the ribs. This coincides with the statement of Dr. Cammann and Dr. Clark.

Dr. Jackson concludes as follows: (1) Auscultatory percussion is of no value in the examination of the liver, the kidney, or the spleen. (2) It is a very valuable method of estimating the area of cardiac dulness, and it is much superior to ordinary percussion for this purpose, but it is not to be regarded as absolutely accurate. (3) It is doubtful if it can be relied upon to delineate less than a quarter of an inch enlargement of the heart, and therefore it is of no value in hypertrophy of the heart without dilatation. (4) By its use the heart may be outlined in conditions of the lung, such as emphysema and consolidation, in which ordinary percussion is not available. (5) If by its use the left border of the heart is recognizable as being outside the areola of the left nipple, or, better, more than four inches and a-half from the midsternal line in a man of medium size, or five inches in a large man, the heart may be considered to be enlarged, especially if the vertical diameter exceeds four inches.

* *N. Y. Med. Jour.*, June 20, 1891.

